# Attenuation of Pain Perception Induced by the Rubber Hand Illusion

**DOI:** 10.3389/fnins.2019.00261

**Published:** 2019-03-22

**Authors:** Wen Fang, Ruyuan Zhang, Yijie Zhao, Liping Wang, Yong-Di Zhou

**Affiliations:** ^1^Key Laboratory of Brain Functional Genomics (MOE and STCSM), Shanghai Changning-ECNU Mental Health Center, Institute of Cognitive Neuroscience, School of Psychology and Cognitive Science, East China Normal University, Shanghai, China; ^2^Center for Magnetic Resonance Research, Department of Neuroscience, University of Minnesota at Twin Cities, Minneapolis, MN, United States; ^3^Institute of Neuroscience, Key Laboratory of Primate Neurobiology, CAS Center for Excellence in Brain Science and Intelligence Technology, Chinese Academy of Sciences, Shanghai, China

**Keywords:** rubber hand illusion, body ownership, laser evoked pain, pain perception, analgesic

## Abstract

Adaptive behavior usually requires accurate representations of body positions and ownership, which rely on integration of multiple sources of sensory information. The rubber hand illusion (RHI) presents a compelling example demonstrating that the combination of visual and tactile signals strongly influences the subjective experience of body ownership. However, it still remains unclear how the perception of body ownership in turn alters other aspects of sensory processing, such as pain perception. In the present study, we examined whether the RHI could modulate the subjective experience of pain. We set three conditions corresponding to different levels of ownership of the rubber hand: the synchronous condition in which the rubber and the real hand were simultaneously stroked; the asynchronous condition in which the two hands were asynchronously stroked; the own-hand-only condition in which only the real hand was stroked. Results from the screening experiment indicated that subjects experienced the stronger RHI in the synchronous condition, compared with the strength of RHI in the other two conditions. In the main experiment, subjects were requested to report the intensity and unpleasantness of pain evoked by laser stimuli under the three stroking conditions. Results showed that pain ratings were significantly lower under the synchronous condition than those under the other two conditions, suggesting the RHI could induce a significant analgesic effect. Furthermore, the correlation analysis showed that the degree of the analgesic effect was positively correlated with the RHI strength across individuals. Taken together, these results suggest an analgesic effect of the RHI and support the potential usage of visual illusions in future translational research on pain.

## Introduction

In our daily life, the sense of body ownership is a fundamental aspect of self-consciousness. The representation of body ownership usually relies on integration of information from multiple sensory modalities (Ehrsson, 2007; Lenggenhager et al., 2007; Blanke et al., 2015). One potent example is the well-studied rubber hand illusion (RHI) (Botvinick and Cohen, 1998). In the RHI experiment, a subject watches a lifelike rubber hand while one of the subject’s real hands is hidden out of sight. The experimenter strokes both the rubber hand and the hidden hand. Strong visual and tactile feedback induces a misperception that the subject feels the rubber hand as his/her own (Ehrsson et al., 2004; Lloyd, 2007).

The illusory feeling of ownership of the rubber hand implies a drastic change in the representation of internal body during the RHI. A bulk of work has shown that the altered body representation in turn produces a range of other consequences in somatosensory and motor processing. For instance, the RHI decreases skin temperature and engenders a tactile dulling effect on the real hand (Moseley et al., 2008a). Subjects experiencing the RHI also tend to underestimate the intensity of touch on their own hand (Kilteni and Ehrsson, 2017). These behavioral consequences are associated with neural changes in the brain. Some studies have reported that during the RHI, a physical threat to the rubber hand evokes comparable cortical startle responses as the threat to the real hand (Ehrsson et al., 2007; Gentile et al., 2013), suggesting that these two hands are subjectively similar. Some other studies have reported that the RHI shifts the topography of the somatosensory homunculus in the primary somatosensory cortex (Schaefer et al., 2009). These results suggest that a change of body ownership in the RHI causes a wide range of perceptual and neural consequences.

In a different line of research, pain perception is a central topic for studying body-related perceptual behavior. The fast and accurate identification of body pain is crucial for humans to protect the body from potential threats. Pain perception is intrinsically associated with the body representation, and therefore it is a reasonable assumption that changes in the body representation alter pain perception (Haggard et al., 2013). It has been shown that visually viewing a body image reduces perceived pain intensity compared to viewing a non-body object (Longo et al., 2009, 2012). Pain intensity is also mediated by the body size, a larger body size inducing a greater reduction in pain intensity (Moseley et al., 2008b). Neuropsychological studies have found similar phenomena. Phantom pain is attenuated if an amputee superimposes his normal limb on the supposed position of his amputated limb in a mirror, a situation where the amputee has an illusory perception of the reappearance of his removed limb (Ramachandran et al., 1995; MacIver et al., 2008; Mercier and Sirigu, 2009; Ramachandran and Altschuler, 2009). A recent study has also shown an analgesic effect of a heartbeat-enhanced virtual reality technology on complex regional pain syndrome (Solca et al., 2018). These examples suggest that pain perception is linked to a range of other aspects of body perception, especially body ownership. Combining those two lines of research, we hypothesized that as a powerful tool to change the representation of body ownership, the RHI would mediate pain perception.

However, such a functional link between the RHI and pain perception has not been firmly established yet. Previous studies addressing this issue fell short in several aspects of experimental design and data analysis. First, it has been known that synchronous stroking to the real and rubber hands is the key to achieve a reliable RHI, whereas asynchronous stroking usually weakens the RHI. If the RHI indeed modulates the perception of pain, distinguishable effects between synchronous and asynchronous stroking conditions should be expected. However, previous studies have failed to find such effects (Hansel et al., 2011; Mohan et al., 2012, but see Hegedus et al., 2014; Martini et al., 2014). Second, no study has established the quantitative link between body ownership and pain perception. It is unclear whether individual differences in the sense of body ownership can predict the variations in pain perception. Third, the effect of the RHI has been shown to be manifested significantly only in 70% of the tested subjects (Kalckert and Ehrsson, 2014). Previous studies usually ignored individual variability and thus the effect of the RHI on pain perception might be diluted due to the weak RHI in a subset of subjects. In addition, a skin-contact thermal pain stimulator has been commonly used to generate pain stimulation in previous studies (Longo et al., 2009), but there are several disadvantages of using it to test the analgesia effect of RHI. The thermal pain stimulator takes a longer time to induce pain perception and may shift subject’s attention from the rubber hand to one’s own hand. Such a distraction may weaken the illusion and reduce the analgesia effect. Furthermore, in order to establish a stronger link between the RHI and pain perception, RHI stimulation and pain stimulation should be spatially and temporally aligned, an aspect that previous studies did not carefully control (Mohan et al., 2012; Hegedus et al., 2014; Martini et al., 2014). Finally, instead of using a fixed-intensity in the stimulation for all subjects, we carefully examined the threshold of pain perception. This approach could avoid the potential floor or ceiling effect because of the considerable individual difference in pain perception (Longo et al., 2009). Taken together, the shortcomings in experimental designs may have hampered previous studies to discover stronger linkage between the RHI and pain perception. It still needs further evidence to demonstrate that illusory ownership of the rubber hand during the RHI indeed alters the perception of pain.

In the present study, we aimed to thoroughly examine the effect of the RHI on pain perception by using an optimized experimental design. We performed a screening experiment to select only subjects who experienced a reliable RHI for the study in main experiments. We also manipulated the strength of the RHI in synchronous, asynchronous and own-hand-only conditions, and quantified pain sensation and unpleasantness in all subjects. In addition, the tactile stimulus in the RHI and the pain stimulus were spatially and temporally adjacent so as to strengthen the interaction between the two stimuli, and thus to strengthen the potential linkage between the RHI and pain perception. We assumed that our efforts would result in an improved study that would maximize the RHI effect on pain perception.

## Materials and Methods

### Ethics Statement

All experimental protocols were approved by the Committee on Human Research Protection at East China Normal University (Approval Letter: HR2013/11003). Informed written consent was obtained from all subjects.

### Subjects

Thirty-three (11 males and 22 females, aged 18–28 years) volunteers were recruited from the community at East China Normal University. Six subjects did not pass the RHI test in the screening experiment due to weak RHI experiences. Twenty-seven subjects (10 males and 17 females, aged 18–28 years) who experienced a robust RHI effect participated in the main experiment. However, three subjects (two males and one female) were excluded from further data analysis: one could not bear the pain stimuli, and the other two rated overly low pain intensity scores (below 15 in a 0 to 100 scale) and reported little or no pain in all stroking conditions.

### The Screening Experiment

Subject screening was conducted before the main experiment to test the strength of the RHI in individual subjects and select subjects who were able to experience a reliable RHI effect. The subject screening included three separate runs corresponding to three stroking conditions (see details below). Each run contained 1-min stroking that was sufficient to induce a robust RHI for most of the subjects. After completing the run, each subject was given a questionnaire, which was the Chinese version of the questionnaire used by Botvinick and Cohen (1998), to measure the subjective strength of the RHI. This questionnaire included two categories of questions (nine questions in total, see Table 1), in which the first three questions were related to the RHI (illusion questions) and the other six questions were control questions. Subjects were requested to rate their subjective experience using a 7-point Likert scale ranged from “−3” (strongly disagree to the statement) to “0” (uncertain) to “+3” (strongly agree to the statement) (Botvinick and Cohen, 1998). When the mean rating score for three illusion questions (Table 1, Q1–Q3) was above 0 (the mean rating score > 0) in a subject, the RHI was considered to have been successfully induced. Thus, only the subjects who had mean scores above 0 in the synchronous condition were selected for the subsequent main experiment.

**Table 1:** Questionnaire of the RHI (Botvinick and Cohen, 1998).

No.	Questions
Q1	It seemed as if I were feeling the touch of the paintbrush in the location where I saw the rubber hand touched.
Q2	It seemed as though the touch I felt was caused by the paintbrush touching the rubber hand.
Q3	I felt as if the rubber hand were my hand.
Q4	It felt as if my hand was drifting toward the rubber hand or arm.
Q5	It seemed as if I might have more than one left hand or arm.
Q6	It seemed as if the touch I was feeling came from somewhere between my own hand and the rubber hand.
Q7	It felts as if my hand was turning ‘rubbery.’
Q8	It appeared as if the rubber hand was drifting toward my hand.
Q9	The rubber hand began to resemble my own hand, in terms of shape, skin tone, freckles or some other visual feature.

### The Main Experiment

**FIGURE 1:**
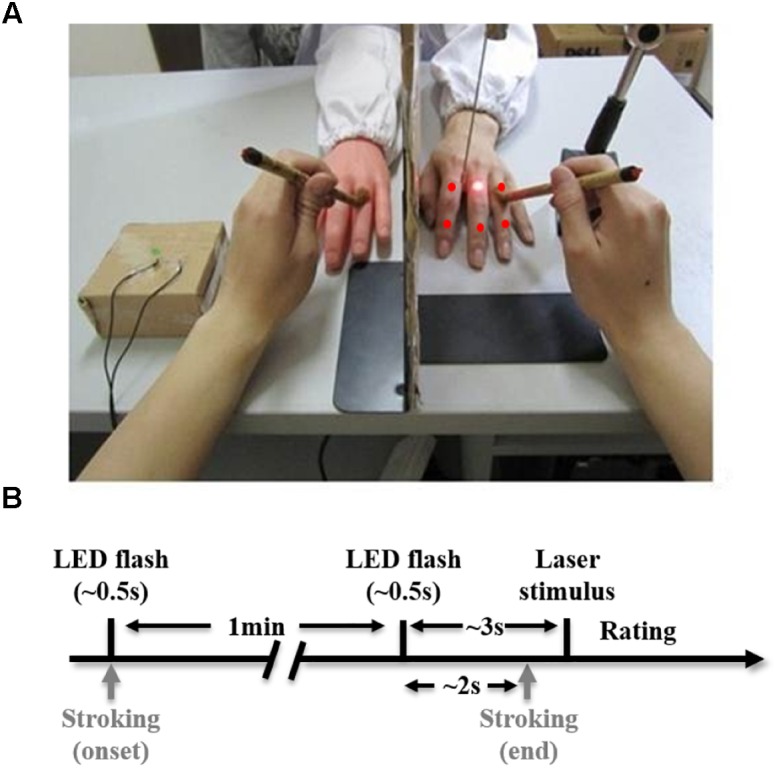
Experimental setup **(A)** and task schematic diagram **(B)**. **(A)** The laser point and five red points indicate six sites where pain stimulation is delivered to the subject’s real hidden hand. **(B)** A trial starts with an LED flash (∼0.5 s), immediately after which, continuous stroking begins. One minute after the onset of stroking, there is a second LED flash (∼0.5 s) indicating an upcoming pain stimulus. About 2 s after the second flash, stroking stops, and about 1 s later, a laser stimulus is delivered to the subject’s real hand. The trial ends after the subject rates pain intensity induced by the laser stimulus.

Subjects were seated with their left arm resting upon a table (Figure 1A). A standing screen was positioned to hide the left arm from view. A lifelike model of the left hand was placed twenty-five centimeters away from the subject’s left hand. The gap between the rubber arm and the subject’s hidden arm was covered by a lab coat to make them more visually comparable. A small LED was positioned near the rubber hand to indicate the onset of a trial and upcoming pain stimulation. Throughout an entire trial, a subject’s eye fixations were held on the dorsal side of the rubber hand (Botvinick and Cohen, 1998).

An experimental trial (in synchronous condition) is illustrated in Figure 1B. We started stroking both the rubber hand and real hand simultaneously and continuously following an LED flash that initiated the trial. About 1 min later, there was another LED flash (the second flash; note that stroking did not stop until 2 s after the onset of this flash.). A pain stimulus (a laser single pulse) was delivered to the dorsum of the real hand 3 s after the second flash. The subject was then required to use the right hand to rate pain intensity, pain unpleasantness, and the RHI strength on a computer. Pain intensity and unpleasantness were rated by moving a cursor on a 100 mm horizontal scale (0–100 scores), anchored from ‘no pain’ to ‘worst imaginable pain’ for intensity and from ‘not unpleasant’ to ‘very unpleasant’ for unpleasantness. To simplify the procedure as there were two more questionnaires included (pain intensity and unpleasantness ratings), we adopted a new version of the rating system, which has been often used in previous studies (Hansel et al., 2011; Mohan et al., 2012; Hegedus et al., 2014). This version was simpler than what we used in the screening experiment. The strength of the RHI was evaluated by a 0 to 10 numeric scale where 0 indicates ‘not felt as if the rubber hand were my hand at all’ and 10 indicates ‘felt very strongly as if the rubber hand were my hand’ (Moseley et al., 2008a). The subsequent trial started immediately after the rating was completed. We used E-Prime 2.0 (Psychology Software Tools, Inc., United States) to present the rating system on the screen and collect behavioral responses.

We set three stroking conditions: synchronous, asynchronous and own-hand-only. In these three conditions, except for the stroking, all other experimental settings were held constant. In the synchronous condition, we simultaneously stroked the real hidden hand and the rubber hand at about 1 Hz. In the asynchronous condition, the hidden hand and the rubber hand were stroked asynchronously at about 1 Hz but different phases (i.e., being stroked alternatively). In both the synchronous and the asynchronous conditions, two identical brushes were used. In the own-hand-only condition, only the real hidden hand was stroked at about 1 Hz, while the rubber hand was not stroked (Lenggenhager et al., 2013; Pazzaglia et al., 2016). Here, the synchronous condition was the standard manipulation to induce the RHI. The asynchronous and own-hand-only conditions acted as controls where the strength of the RHI were significantly reduced.

The experiment consisted of a practice run and three subsequent experimental runs in line with the three stroking conditions. The practice run was always the first run and the order of the three experimental runs was random and counterbalanced across subjects. Each run included six trials corresponding to the six stimulation sites on the hand (Figure 1A). The order of six stimulation positions was also randomized within each run. In the practice run, subjects were instructed to hold fixations on the rubber hand for 1 min. Neither the real hand nor the rubber hand was stroked. For three experimental runs, the hands were stroked for 1 min.

### Pain Stimuli

Pain stimuli were delivered by a non-contact infrared laser (StarMedTec GmbH). Before the experiment, we calibrated the strength of the laser pulse for each individual subject using a method of adjustment (Longo et al., 2009). This calibration involved a series of pain stimuli delivered from 160 to 480 mJ with a 20 mJ step, then in a reversed order from 480 to 160 mJ with the same step size. Such testing circle was repeated twice. Subjects verbally reported subjective pain intensity by choosing a number from 0 (no pain) to 10 (worst imaginable pain). Laser intensity readings corresponding to the score of 5 were averaged and this average intensity was used as stimulus intensity in the experiment for that subject. The mean of calibrated intensity cross all subjects was 360.74 mJ and the standard error was 9.12 mJ (*n* = 27, range from 240 to 440 mJ).

### Data Analysis

Behavioral data were analyzed using the SPSS statistical software package (version 20.0, IBM Corp., United States). In the screening experiment, for each subject we averaged scores for three illusion questions and scores for six control questions. A 3 × 2 two-way repeated-measures ANOVA was then performed with the RHI rating score as the dependent variable, question categories (illusion/control questions) and three stroking conditions (synchronous/asynchronous/own-hand-only) as within-subject variables.

In the main experiment, we undertook a one-way repeated-measures ANOVA (factor: stroking condition with three levels) on each respective type of rating (pain intensity, unpleasantness and illusion strength). All variables in both subject screening and main experiments were normally distributed according the Kolmogorov–Smirnov tests with Lilliefors correction (all *p*s > 0.05). All pairwise *post hoc* comparisons in ANOVA analyses were Bonferroni corrected. Significance was set at α = 0.05. In the correlation analysis, we normalized relative pain intensity and unpleasantness in the synchronous condition against the asynchronous condition:

(1) $$\mathrm{Intensity reduction index}=(P_{\mathrm{async}}-P_{\mathrm{sync}})/(P_{\mathrm{async}}+P_{\mathrm{sync}})$$

(2) $$\mathrm{Unpleasantness reduction index}=(U_{\mathrm{async}}-U_{\mathrm{sync}})/(U_{\mathrm{async}}+U_{\mathrm{sync}})$$

where *P_sync_* and *P_async_* indicate rating scores of pain intensity under synchronous and asynchronous conditions, respectively, and similar conventions are applied to the unpleasantness rating. Here, both the pain intensity reduction index and unpleasant reduction index reflect the degree of rating score reduction in the synchronous condition compared to the asynchronous condition, with a larger value indicating a stronger analgesic effect. Similarly, the strength of the RHI was computed as:

(3) $$\mathrm{Illusion strength index}=(I_{\mathrm{sync}}-I_{\mathrm{async}})/(I_{\mathrm{async}}+I_{\mathrm{sync}})$$

where *I_sync_* and *I_async_* indicate RHI rating scores under synchronous and asynchronous conditions, respectively. A larger illusion strength index indicates a stronger RHI effect in the synchronous condition compared to the asynchronous condition.

## Results

### Reliable RHI Effects Induced by Synchronously Stroking in the Subject Screening Experiment

**FIGURE 2:**
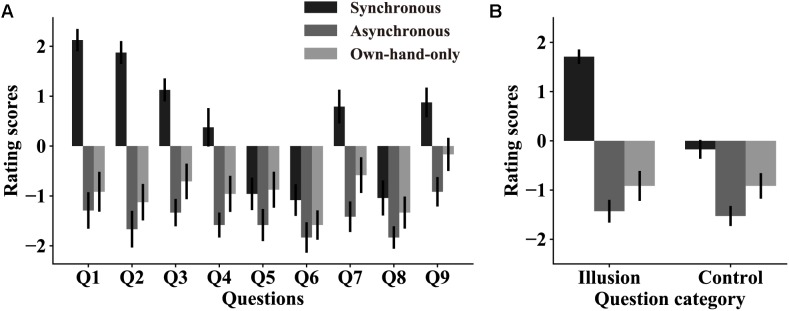
The RHI strength in subject screening. **(A)** Rating scores on all nine questions in three stroking conditions. Error bars represent SEMs across subjects. All nine questions can be classified into two categories: illusion questions (Q1–Q3) and control questions (Q4–Q9). **(B)** Averaged rating scores on illusion questions and control questions in each stroking condition. RHI strength scores are higher in the synchronous condition than the other two conditions. These results suggest that our stroking manipulation indeed produces significant RHI effects in the synchronous condition but not in the other two conditions.

Three stroking conditions were set to induce the RHI at different levels of strength. This was critical since if the RHI indeed altered pain perception, such modulation effects should vary with changes in RHI strength. We first assessed whether this manipulation was effective in subject screening, which we also used to select subjects who could be induced a strong RHI (see section “Materials and Methods”). Out of 33 subjects, 27 passed the screening phase. However, only 24 subjects (out of the 27) were used in the analysis as three more subjects who failed to rate their pain perception in the following main experiment were also excluded (see section “Materials and Methods”). Figure 2A shows that there was a main effect of the question categories [illusion question: 1.7 ± 0.14, synchronous; −1.4 ± 0.23, asynchronous; −0.9 ± 0.30, own-hand-only. control question: −0.2 ± 0.19, synchronous; −1.5 ± 0.20, asynchronous; −0.9 ± 0.26, own-hand-only, *F*(1,23) = 28.26, *p* < 0.0001, η^2^ = 0.55], indicating that the subjects experienced an overall vivid illusion in subject screening. A main effect of the stroking conditions [*F*(2,46) = 51.07, *p* < 0.0001, η^2^ = 0.69] was also observed, which was substantiated using pairwise comparisons across three stroking conditions. This result revealed that the three stroking conditions indeed created different strength levels of the RHI. Figure 2B shows that the RHI was stronger in the synchronous condition than in the asynchronous condition (*p* < 0.0001) or own-hand-only (*p* < 0.0001) condition, while there was no significant difference between the later two conditions (*p* = 0.46). More importantly, a significant interaction between the question category and the stroking condition was noted [*F*(2,46) = 26.62, *p* < 0.0001, η^2^ = 0.54]. Pairwise comparisons showed that the average score for illusion questions was significantly higher than that for control questions in the synchronous condition (*p* < 0.0001), but such differences were not significant in both asynchronous (*p* = 0.61) and own-hand-only conditions (*p* > 0.999). Taken together, these results suggested a reliable RHI effect in the synchronous condition, which was much stronger than that in the asynchronous condition or the own-hand-only condition. These results are also in line with a prior work (Botvinick and Cohen, 1998).

### RHI Rating Results in the Main Experiment

**FIGURE 3:**
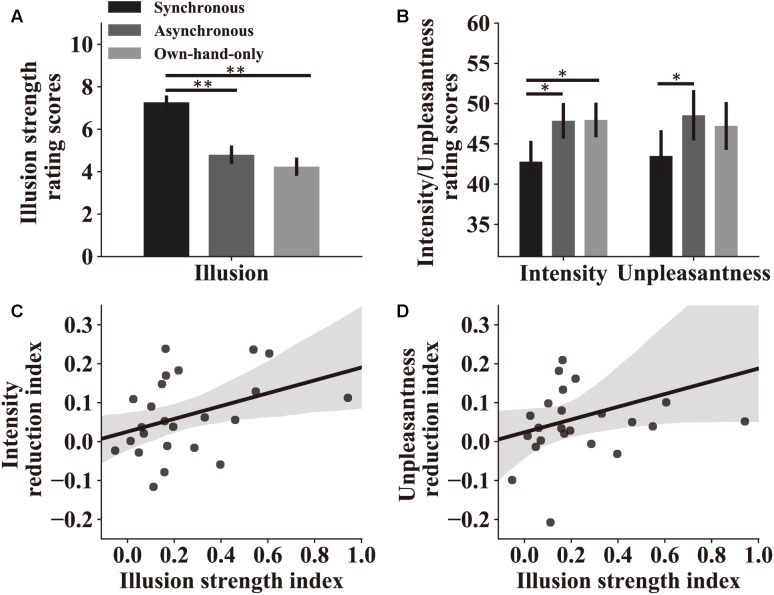
Attenuation by the RHI on perceived pain intensity and unpleasantness. **(A)** Rating scores of the RHI strength in the main experiment. Results indicate that three stroking conditions indeed produce different levels of the RHI. **(B)** Rating scores of pain intensity and unpleasantness in the three stroking conditions. Both scores reflect the level of perceived pain, which is lower in the synchronous condition compared to the other two conditions. For both **(A,B)**, significant symbol conventions are ^∗^*p* < 0.05; ^∗∗^*p* < 0.01. **(C)** Pain intensity reduction index reflects the strength of the analgesic effect in the synchronous condition compared to the asynchronous condition. A larger value represents a stronger analgesic effect (see section “Materials and Methods”). Pain intensity reduction indices of individual subjects are significantly correlated with their illusion strength indices (*r* = 0.42, *p* = 0.040), which reflect the difference of the RHI strength in two stroking conditions (see section “Materials and Methods”). **(D)** Pain unpleasant reduction indices and illusion strength indices show a trend in correlation analysis at the individual level (*r* = 0.33, *p* = 0.11) similar to the analysis of pain intensity. For both **(C,D)**, the shaded area represents the 95% confidence interval for correlations.

Similar to what we did in the subject screening experiment, in the main experiment we examined the overall strength of the RHI and the effectiveness of our stroking manipulation. Subjects were instructed to rate the RHI strength using a 0–10 numeric scale (see details in section “Materials and Methods”). A one-way repeated-measure ANOVA was performed with the RHI strength score as the dependent variable, and stroking conditions as the within-subject factor. Results replicated findings in subject screening and showed a main effect of stroking conditions [synchronous, 7.3 ± 0.32; asynchronous, 4.8 ± 0.44, own-hand-only, 4.2 ± 0.43, *F*(2,46) = 30.82, *p* < 0.0001, η^2^ = 0.57, Figure 3A]. A pairwise comparison analysis further showed that the subjects experienced a stronger RHI in the synchronous condition than in the asynchronous condition (*p* < 0.0001) or the own-hand-only condition (*p* < 0.0001). No significant difference in the RHI was observed between the later two conditions (*p* = 0.48).

### Attenuation of Pain Perception Induced by the RHI

We next turned to the main focus of our study: whether the RHI mediated perceived pain and how such an effect differed across stroking conditions.

We then assessed how pain perception was altered in the experiment. All subjects were requested to rate pain intensity and unpleasantness after experiencing pain stimuli. Two repeated-measure ANOVAs were performed with perceived pain intensity or unpleasantness as the dependent variable, and stroking conditions as the independent variable. We observed significant main effects of stroking conditions on both pain intensity [synchronous, 42.8 ± 2.58; asynchronous, 47.9 ± 2.20, own-hand-only, 48.0 ± 2.15, *F*(2,46) = 7.45, *p* = 0.002, η^2^ = 0.25, Figure 3B] and unpleasantness [synchronous, 43.5 ± 3.19; asynchronous, 48.5 ± 3.13, own-hand-only, 47.2 ± 2.97, *F*(2,46) = 5.28, *p* = 0.009, η^2^ = 0.19, Figure 3B]. Pairwise comparisons revealed that rating scores on pain intensity in the synchronous condition were significantly lower than those in the asynchronous condition (*p* = 0.016) or the own-hand-only condition (*p* = 0.013), whereas no significant difference was discovered between the later two conditions (*p* = 0.935). Similarly, rating scores on unpleasantness in the synchronous condition were significantly lower than those in the own-hand-only condition (*p* = 0.030), and marginally lower than those in the asynchronous condition (*p* = 0.056). There was no difference in unpleasantness scores between asynchronous and own-hand-only conditions (*p* > 0.999). These results provide direct support for our hypothesis that synchronous stroking induces a stronger RHI effect which in turn attenuates perceived pain intensity and unpleasantness.

### Correlations Between RHI Strength and Pain Intensity Reduction

Correlation analyses were performed to examine the link between the RHI strength and the degree of pain reduction across all individual subjects. For each subject, we calculated an illusion strength index to quantify the relative strength of the RHI in the synchronous condition compared to the asynchronous condition. We also calculated a pain intensity reduction index and an unpleasant reduction index to quantify the difference in perceived pain between synchronous and asynchronous conditions (i.e., quantifying the analgesic effect). A Spearman’s rank-order correlation analysis showed a significant positive relationship between the illusion strength index and the pain intensity reduction index (*r* = 0.42, *p* = 0.040), revealing that the stronger RHI produces the stronger analgesic effect (Figure 3C). A similar trend was also observed between illusion strength and unpleasantness reduction (*r* = 0.33, *p* = 0.11, Figure 3D).

## Discussion

Despite long-hypothesized theoretical links between the RHI and pain perception, there is little empirical research directly supporting those links (Mohan et al., 2012). By carefully optimizing several aspects of experimental design, our study showed that the RHI could effectively attenuate perceived pain. That is, ratings of pain intensity and unpleasantness were significantly reduced in the synchronous condition where the strongest RHI was observed among three experimental conditions. A further analysis found that there was a significant correlation between the strength of the RHI and the reduction in pain intensity across individual subjects. Our current results shed a new light on the analgesic effect induced by the RHI, which has been hypothesized by previous studies (Hansel et al., 2011; Hegedus et al., 2014; Martini et al., 2014; Siedlecka et al., 2014; Pozeg et al., 2017).

One main result in our study is that we found altered pain perception between synchronous and asynchronous stroking conditions. To validate the analgesic effect of the RHI, the key is to set up an appropriate baseline condition, otherwise additional confounding factors may be introduced. For example, some studies set the control condition by replacing the rubber hand with a body-irrelevant non-corporeal object, and contrasted pain perception by synchronously stroking the subject’s hand and non-corporeal object (Hansel et al., 2011; Martini et al., 2014). Using a non-corporeal object might be debatable since it looks drastically different from a hand and such visual discrepancy itself may alter perceived pain (Moseley et al., 2008b; Younger et al., 2010; Eisenberger et al., 2011). It is therefore unclear whether pain reduction observed in those studies should be attributed to a change in body ownership or a difference in visual appearance. In our study, we used asynchronous stroking as the control condition and this manipulation ensured to weaken the strength of the RHI with no change on other experimental settings. We also used the own-hand-only condition as another baseline in which no visual feedback was provided, thereby further abolishing the potential RHI effect. The own-hand-only can serve as a ‘ground truth’ to examine the efficacy of asynchronous stroking. One prior study found that there was a difference between the synchronous and the asynchronous conditions (Hegedus et al., 2014). However, that study did not compare the asynchronous condition with a well-controlled ‘ground truth.’ Similary, in Mohan et al. (2012) study, synchronous and asynchronous conditions were arranged pseudorandomly within a block. It’s possible that the post effect of the synchronous trial inceased the strength of illusion in the next asynchronous trial (Hohwy and Paton, 2010). Furthermore, instead of measuring the illusion strength after each trial, the authors incorporated two separate stroking sessions (synchronous and asynchronous), in which, at the end of the experiment, there was no pain stimulus to evaluate the illusion strength. Therefor it was difficult to estimate the illusion strength in those asynchronous trials. Here, our results showed that asynchronous strokes did not alter perceived pain, as evidenced by comparable RHI rating scores between asynchronous and own-hand-only conditions. Thus, the difference between synchronous and asynchronous conditions in our study was most likely because of pain *reduction* in the synchronous condition rather than pain *enhancement* in the asynchronous condition. We believe that our study was carefully designed to minimize those confounding issues.

Another main result in the current study is that we found significant correlation between pain reduction and RHI strength at the individual level. That is, a stronger RHI produces a stronger analgesic effect (Figure 3C). This finding, together with the finding of alteration of pain perception between stroking conditions, strongly support the assumption that the RHI attenuates perceived pain and this effect is induced primarily by the illusion of body ownership.

We speculate that three factors may contribute to the analgesic effect.

First, the core feature of the RHI is the acquired ownership of the rubber hand, and this may act as the primary cause for pain reduction. Previous studies have shown that viewing the body part where pain stimuli are applied reduces perceived pain (Longo et al., 2009, 2012). Likewise, in the RHI, if a subject erroneously feels the rubber hand that is visiable as his/her own hand, it may theoretically be equivalent to the condition in which the subject’s own hand is visible, thereby reducing pain. However, in the present study, the real hand in both own-hand-only and asynchronous conditions was blocked. Thus, there was no difference between the two conditions. This explanation suggests that the analgesic effect largely depends on the extent of illusory ownership of the rubber hand, which is consistent with our correlation analysis (Figure 3C,D).

Second, disownership of the real hand may also contribute to the altered pain perception. Disownership of the real hand and acquired ownership of the rubber hand are ostensibly equivalent but some previous studies have suggested that they manifest two independent processes (Gentile et al., 2013; Kilteni and Ehrsson, 2017). One example is the “third-hand” phenomenon – namely, subjects may feel that both the hidden real hand and the rubber hand belong to themselves (Schaefer et al., 2009). This phenomenon suggests that illusory ownership of the rubber hand is not necessarily accompanied by disownership of the real hand. Thus, it is also possible that the later one induces the analgesic effect (Moseley et al., 2008a; Hegedus et al., 2014; Martini et al., 2014; Kilteni and Ehrsson, 2017; Pozeg et al., 2017). Future research is needed to further elaborate on this issue.

Third, pain reduction may stem from conflicts between visual feedback and tactile feedback during the RHI. That is, information that induces the RHI is from different sensory channels and different spatial locations, visual information coming from the rubber hand and tactile information coming from the hidden real hand. Such conflicts between information sources may reduce pain. For example, a study has found that perceived pain is attenuated if the spatial reference of the body is disrupted (Gallace et al., 2011). Furthermore, some studies have found that the perceived position of the real hand shifts to the rubber hand in the RHI (Botvinick and Cohen, 1998; Ehrsson et al., 2005; Costantini and Haggard, 2007; Longo et al., 2008), and such mislocalization of the real hand is independent of the feeling of ownership of the rubber hand (Holle et al., 2011; Abdulkarim and Ehrsson, 2016). These results suggest that spatial information of body parts is important for pain perception and may act as a process that is independent of body ownership. In a recent study, Siedlecka et al. (2018) have found that pain ratings are increased in the illusion condition accompanied by a change in the perceived location of pain, when the rubber hand is invisible. Instead, our results demonstrated that the perception of pain significantly decreased if the rubber hand was visible, suggesting the importance of the visible hand from either the real own hand or the rubber hand perceived as one’s own in the analgesic effect. Existing studies using the RHI, however, have not demonstrated a direct relationship between limbs localization and pain reduction. Future research therefore is needed to further elucidate such relationship.

To maximize the effect of the RHI on pain perceptiom, we carefully optimized several aspects of experimental design in the current study.

First, existing work has shown that only 70% of subjects who are able to experience a reliable RHI effect (Ehrsson et al., 2005; Kalckert and Ehrsson, 2014). Given that our goal is to test the effect of the RHI on pain perception, a prerequisite is to produce a robust RHI. The inclusion of 30% of subjects who are not able to experience the RHI may weaken the statistical power, and thereby draw a false conlusion that the RHI analgesic effect is not significant. In the current study, we conducted a subject screening experiment to (1) test the efficacy of stroking manipulation, and (2) select subjects who experienced a reliable RHI. According to this subject screening, 27 out of 33 subjects reported a significant RHI, a ratio (∼81%) similar to previous studies (Ehrsson et al., 2005; Kalckert and Ehrsson, 2014). To ensure a robust test of the effect of RHI on pain perception, only those 27 subjects were selected for the current study.

Second, we used a non-contact infrared laser stimulator rather than a skin-contact thermal pain stimulator to generate pain stimulation. Using the infrared laser stimulator could avoid several confounding issues. For example, if a thermal stimulator had been used, it should have been stabilized on both hands to ensure that the rubber hand and the real hand were both visually and tactilely comparable (Mohan et al., 2012). However, there is evidence showing that visually viewing a harmful object (i.e., the thermal stimulator) may also alter pain perception (Valeriani et al., 2008; Hofle et al., 2012). In our study, we only placed the laser stimulator for the real hand, which was invisible as being hidden by the standing screen, thereby eliminating potential confounding visual information. In addition, in previous studies (Mohan et al., 2012; Hegedus et al., 2014; Martini et al., 2014), the thermal pain stimulator may introduce additional tactile sensation as it has to be directily attached to both the fake hand subject’s own hand. Such tactile sensation itself may act as synchronous stimulation, reducing the difference in the illusion strength between experimental conditions.

Third, the use of a laser stimulator in our study had several other advantages. As shown in some previous studies (Mohan et al., 2012; Hegedus et al., 2014; Martini et al., 2014), if a thermal stimulator had been attached to the real hand in our study, the stroking site would have been forced to deviate from the pain stimulation site where the thermal stimulator had been placed. Such spatial displacement may weaken the illusion and thus reduce the analgesia effect. By using the laser stimulator, however, we could stroke as well as apply pain stimulation to the same site on the real hand, and thereby avoid the spatial displacement. This resulted in a stronger link between the RHI and pain perception compared to the other studies (Mohan et al., 2012; Hegedus et al., 2014; Martini et al., 2014). In addition, we changed the location of pain stinulation in every trial to prevent an increased sensitivity to pain in the subject. However, if a skin-contact thermal pain stimulator had been used in our study, such a change in location of pain stinulation would have been unrealistic, since it would be difficult to relocate the thermal pain stimulator trial by trial. Furthermore, compared to a laser stimulator, a thermal pain stimulator takes a longer time to induce pain, the subject’s attention is thus more likely to shift from the rubber hand to his/her own hand. Such a shift may also reduce the analgesia effect.

Finally, we carefully measured the threshold of stimulation strength (see section “Materials and Methods”) in each subject and used this threshold to determine individual intensity of pain stimuli in the main experiment. This manipulation is consistent with a previous study (Longo et al., 2009). This approach, compared to the approach of using fixed intensity of simulation for all subjects, can avoid the potential floor or ceiling effect because of the considerable individual difference in pain perception. This approach may also diminish the impact of attention or arousal when pain stimulation is too strong to some subjects.

In summary, our results indicate that the RHI attenuates perceived pain intensity and unpleasantness, and this analgesic effect is positively correlated with the RHI. Our results provide empirical support for the relationship between body ownership and pain perception, which may advance our understanding of the mechanisms of body pain and promote pain rehabilitation in the future. Also, the functional link between the analgesic effect and the extent of illusory body ownership suggests that methods such as perceptual illusions, virtual reality, or the utilization of multisensory information may confer analgesic consequences in future clinical practice.

## Data Availability

Data are available from the corresponding author upon reasonable request.

## Author Contributions

Y-DZ, LW, and WF conceived and designed the study. WF and YZ collected and analyzed the data. Y-DZ, RZ, and WF wrote the manuscript.

## Conflict of Interest Statement

The authors declare that the research was conducted in the absence of any commercial or financial relationships that could be construed as a potential conflict of interest.
